# Genome Sequence of Mycobacterium Phage Guppsters

**DOI:** 10.17912/micropub.biology.001450

**Published:** 2025-02-17

**Authors:** Emma K Alley, Lexi C Hill, Mya E Houglum, Maggie R Lamppa, Khailee K Pack, Hailey D Pageau, Cale J Prosen, Kylie R Richards, Kendra S Royer, Emily A Slettedahl, Ian L Strusz, Lydia A Wiita, Jillian C Zeidler, Daniel E Westholm

**Affiliations:** 1 College of St. Scholastica, Duluth, Minnesota, United States

## Abstract

Mycobacterium Phage Guppsters was isolated on *Mycobacterium smegmatis* mc ^2^ 155, displays a siphovirus morphology, and possesses a 54,835 base pair genome. Based on gene content similarity, Guppsters is assigned to cluster F1. Unlike a majority of F1 phages, Guppsters does not encode mycobacteriophage mobile elements.

**Figure 1. Guppsters virion morphology:**
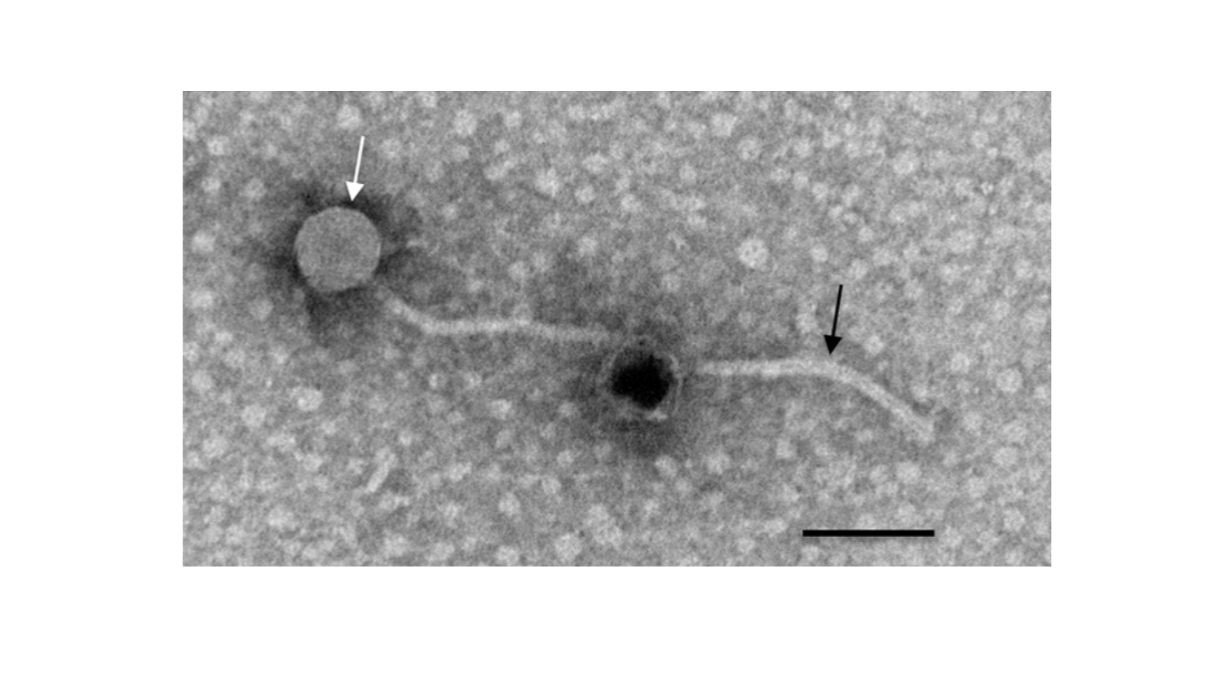
Transmission electron micrograph of mycobacteriophage Guppsters negative stained with phosphoric tungsten acid displaying siphovirus morphology with isometric capsid (70 nM, white arrow) and flexible tail (200 nM, black arrow)(n=1). Scale bar is 100 nm.

## Description

The genus *Mycobacterium* contains many disease-causing pathogens, with some establishing antibiotic-resistant infections. *Mycobacterium smegmatis* is a non-pathogenic and genetically tractable member of the genus, and bacteriophages isolated on *M. smegmatis* have been used to treat antibiotic-resistant mycobacterial infections (Barka et al., 2016; Dedrick et al., 2019) . Here, we report the discovery and characteristics of a novel mycobacteriophage, Guppsters, isolated using *Mycobacterium smegmatis* mc ^2^ 155.

Guppsters was isolated from potted plant soil from the green roof of The College of St. Scholastica Science Building in Duluth, Minnesota (GPS coordinates: 46.816111 N, 92.104444 W). The soil sample was suspended in Middlebrook 7H9 liquid media, inoculated with *M. smegmatis* mc ^2^ 155 and incubated at 37 ^o^ C with 250 rpm shaking for 24 hours to enrich for mycobacteriophages. Following enrichment, the sample was vacuum-filtered (0.22 µM pore size), the filtrate was spotted on top agar supplemented with *M. smegmatis,* and the plates incubated at 37˚C for 48 hours, yielding a clearing on the bacterial lawn by bacteriophage Guppsters. Guppsters was purified through three rounds of plating for plaques. After purification, a liquid lysate was prepared and used for negative staining transmission electron microscopy using 1% phosphoric tungsten acid, revealing a siphovirus morphology with a 70 nm isometric capsid and 200 nm tail (n=1) (Russell and Hatfull, 2017) .

DNA was isolated from the liquid lysate using the Promega Wizard DNA cleanup kit. Sequencing of Guppsters DNA was completed with an Illumina MiSeq (v3 reagents) using a library prepared with NEB Ultra II Library Kit, producing 392,678 single-end 150-base reads, representing ~1,000-fold coverage. Newbler v2.9 (Silva et al., 2013) and Consed v29 (Gordon and Green, 2013) were used to assemble the raw reads and verify completeness, using default parameters. The 54,835 base pair Guppsters genome contains 3’ single stranded overhang (5’CGGTAGGCGC). Its GC content of 62.47% GC is similar to its isolation host *M. smegmatis* ( https://www.ncbi.nlm.nih.gov/nuccore/CP000480.1 ).

Guppster’s genome was auto-annotated using Glimmer (Delcher et al., 2007) and GeneMark (Besemer and Borodovsky, 2005) , then refined through manual annotation with DNA Master ( http://cobamide2.bio.pitt.edu ), PECAAN ( http://discover.kbrinsgd.org ), Phamerator (Cresawn et al., 2011) , Starterator ( http://phages.wustl.edu/starterator/ ), BLAST (Altshul et al., 1990), and HHPRED (Soding et al., 2005) . Aragorn v1.2.41 (Laslett and Canback, 2004) and tRNAscan-SE v2.0 (Lowe and Eddy, 1997) detected no tRNA genes. Databases accessed include the following: BLAST-Actinobacteriophage and NCBI non-redundant database; HHPRED- PDB_mmCIF70, Pfam v36, NCBI Conserved Domain Database v3.20; and Phamerator- Actino_draft database v578. Guppsters is assigned to cluster FI based on gene content similarity of at least 35% to phages in the Actinobacteriophage database, phagesdb ( https://phagesDB.org ) (Russell and Hatfull, 2017) . All software were used with default parameters.

As with other cluster F1 phages, Guppsters encodes an immunity repressor and a tyrosine integrase, suggesting it is able to establish lysogeny. Guppster also encodes Cro (control of repressor’s operator) adjacent to its immunity repressor gene. A majority of cluster F1 phages (154/240 phages, to date), encode mycobacteriophage mobile elements (MPMEs) (Sampson et al., 2009) . Interestingly, annotation failed to identify MPMEs in Guppster’s genome, despite the presence of a putative MPME 2 in LittleShirley (Russell and Hatfull, 2017) , another F1 phage isolated from the same green roof potted plant as Guppsters. Guppsters and LittleShirley share 58% gene content similarity (Russell and Hatfull, 2017) .

**Nucleotide sequence accession numbers**

Guppsters is available at GenBank with Accession No. PP978892 and Sequence Read Archive (SRA) No. SRX25734229.
